# The AgingPLUS Trial: Intervening to Promote Physical Activity in Middle-Aged and Older Adults

**DOI:** 10.1093/geroni/igab046.1683

**Published:** 2021-12-17

**Authors:** David Roth, Shang-En (Michelle) Chung, Kaigang Li, Abigail Nehrkorn-Bailey, Katherine Thompson, Manfred Diehl, George Rebok

**Affiliations:** 1 Johns Hopkins University, Baltimore, Maryland, United States; 2 Colorado State University, Fort Collins, Colorado, United States

## Abstract

This paper investigated whether the AgingPLUS program promotes physical activity in middle-aged and older adults by examining outcomes at weeks 4 and 8 with baseline scores included as covariates. The analyses assessed intervention effects on negative views of aging (NVOA), physical activity (CHAMPS), physical function (SPPB, VO2max), and accelerometry measures (e.g., minutes sedentary). We found significant intervention effects on NVOA (p < .001) and frequency of moderate intensity exercise (p = 0.048), but no significant effects on physical function, VO2max, or the accelerometry measures. Standardized effect sizes for the significant effects ranged from 0.31 to 1.03 standard deviation units. These findings suggest that AgingPLUS improved motivational factors for engaging in physical activity but did not lead to objective changes in physical activity in the short term. Further research will investigate the mediational role of these motivational factors in enhancing physical activity over the longer term (6 months).

